# Prognostic Factors for Overall Survival In Chronic Myeloid Leukemia Patients: A Multicentric Cohort Study by the Italian CML GIMEMA Network

**DOI:** 10.3389/fonc.2021.739171

**Published:** 2021-08-26

**Authors:** Giorgina Specchia, Patrizia Pregno, Massimo Breccia, Fausto Castagnetti, Chiara Monagheddu, Massimiliano Bonifacio, Mario Tiribelli, Fabio Stagno, Giovanni Caocci, Bruno Martino, Luigiana Luciano, Michele Pizzuti, Antonella Gozzini, Anna Rita Scortechini, Francesco Albano, Micaela Bergamaschi, Isabella Capodanno, Andrea Patriarca, Carmen Fava, Giovanna Rege-Cambrin, Federica Sorà, Sara Galimberti, Monica Bocchia, Gianni Binotto, Giovanni Reddiconto, Paolo DiTonno, Alessandro Maggi, Grazia Sanpaolo, Maria Stella De Candia, Valentina Giai, Elisabetta Abruzzese, Maria Cristina Miggiano, Gaetano La Barba, Giuseppe Pietrantuono, Anna Guella, Luciano Levato, Olga Mulas, Fabio Saccona, Gianantonio Rosti, Pellegrino Musto, Francesco Di Raimondo, Fabrizio Pane, Michele Baccarani, Giuseppe Saglio, Giovannino Ciccone

**Affiliations:** ^1^Former Full Professor of Hematology- University of Bari Aldo Moro” Bari GIMEMA WP CML, Bari, Italy; ^2^Haematology Unit, Azienda Ospedaliero-Universitaria Città della Salute e della Scienza, Torino, Italy; ^3^Department of Cellular Biotechnologies and Hematology, Sapienza University of Rome, Roma, Italy; ^4^Department of Experimental, Diagnostic and Specialty Medicine, S. Orsola-Malpighi Hospital, University of Bologna, Bologna, Italy; ^5^Clinical Epidemiology Unit and CPO Piemonte, Città della Salute e della Scienza, Torino, Italy; ^6^Section of Hematology, Department of Medicine, University of Verona, Verona, Italy; ^7^Division of Hematology and BMT, Department of Medical Area, University of Udine, Udine, Italy; ^8^Division of Hematology and Bone Marrow Transplant, Azienda Ospedaliera-Universitaria (AOU) Policlinico-V. Emanuele, Catania, Italy; ^9^Department of Medical Sciences and Public Health, Businco Hospital, University of Cagliari, Cagliari, Italy; ^10^Haematology Unit, Azienda Ospedaliera “Bianchi-Melacrino-Morelli”, Reggio Calabria, Italy; ^11^Haematology Unit “Federico II”, University of Naples, Naples, Italy; ^12^Department of Hematology, “San Carlo” Regional Hospital, Potenza, Italy; ^13^Haematology Unit, AOU Careggi, University of Florence, Florence, Italy; ^14^Division of Hematology, Department of Molecular and Clinical Sciences, Polytechnic University of Marche, Ancona, Italy; ^15^Department of Emergency and Transplantation, Hematology Section, University of Bari Medical School, Bari, Italy; ^16^Clinical Hematology, Policlinico San Martino, Genua, Italy; ^17^Department of Hematology, Azienda UNITà SANITARIA LOCALE (USL)-IRCCS di Reggio Emilia, Viale Risorgimento, Reggio Emilia, Italy; ^18^Division of Hematology, Department of Translational Medicine, University of Eastern Piedmont, Novara, Italy; ^19^Department of Clinical and Biological Sciences, University of Turin, Turin, Italy; ^20^Orbassano Hospital, Turin University, Turin, Italy; ^21^Institute of Hematology, Università Cattolica Sacro Cuore, Rome, Italy; ^22^Department of Clinical and Experimental Medicine, Unità Operativa (UO) Haematology, AOU Pisana, Pisa, Italy; ^23^Haematology Unit, Azienda Ospedaliera Universitaria Senese, University of Siena, Siena, Italy; ^24^Padova Hematology and Clinical Immunology, Padua, Italy; ^25^Department of Ematologia, Lecce Ematologia Ospedale Vito Fazzi, Lecce, Italy; ^26^Haematology Unit, National Cancer Center, IRCCS Istituto Tumori “Giovanni Paolo II”, Bari, Italy; ^27^Division of Hematology, Hospital “S.G. Moscati”, Taranto, Italy; ^28^Department of Hematology and Stem Cell Transplantation Unit, IRCCS Casa Sollievo della Sofferenza Hospital, San Giovanni Rotondo, Italy; ^29^Hematology, Hospital A. Perrino, Brindisi, Italy; ^30^Hemoglobinopathies Unit, Hematology Department, S. Eugenio Hospital (ASL Roma 2), Rome, Italy; ^31^Hematology Department, San Bortolo Hospital, Vicenza, Italy; ^32^Department of Hematology, “Spirito Santo” Hospital, Pescara, Italy; ^33^Hematology Oncology, IRCCS Centro di Riferimento Oncologico della Basilicata, Rionero in Vulture, Italy; ^34^Hematology Unit, Santa Chiara Hospital, Trento, Italy; ^35^Haematology Unit, A. Pugliese Hospital, Azienda Ospedaliera Pugliese Ciaccio, Catanzaro, Italy

**Keywords:** chronic myeloid leukemia, tyrosine kinase inhibitors, prognostic factors, ELTs, Sokal score

## Abstract

An observational prospective study was conducted by the CML Italian network to analyze the role of baseline patient characteristics and first line treatments on overall survival and CML-related mortality in 1206 newly diagnosed CML patients, 608 treated with imatinib (IMA) and 598 with 2^nd^ generation tyrosine kinase inhibitors (2GTKI). IMA-treated patients were much older (median age 69 years, IQR 58-77) than the 2GTKI group (52, IQR 41-63) and had more comorbidities. Estimated 4-year overall survival of the entire cohort was 89% (95%CI 85.9-91.4). Overall, 73 patients (6.1%) died: 17 (2.8%) in the 2GTKI vs 56 (9.2%) in the IMA cohort (adjusted HR=0.50; 95% CI=0.26-0.94), but no differences were detected for CML-related mortality (10 (1.7%) vs 11 (1.8%) in the 2GTKIs vs IMA cohort (sHR=1.61; 0.52-4.96). The ELTS score was associated to CML mortality (high risk vs low, HR=9.67; 95%CI 2.94-31.74; p<0.001), while age (per year, HR=1.03; 95%CI 1.00-1.06; p=0.064), CCI (4-5 vs 2, HR=5.22; 95%CI 2.56-10.65; p<0.001), ELTS score (high risk vs low, HR=3.11; 95%CI 1.52-6.35, p=0.002) and 2GTKI vs IMA (HR=0.26; 95%CI 0.10-0.65, p=0.004) were associated to an increased risk of non-related CML mortality. The ELTS score showed a better discriminant ability than the Sokal score in all comparisons.

## Introduction

The treatment landscape of patients with chronic myeloid leukemia (CML) changed dramatically after the approval of imatinib, the first tyrosine kinase inhibitor (TKI), in 2001 (1). Since then, several newer TKIs have also been approved, and 3 different TKIs are currently available as front-line treatments in Italy (2). Due to the remarkable efficacy of TKIs therapy, the life expectancy of newly diagnosed chronic phase (CP) patients is now near to that of age-matched individuals in the general population (3). The improved outcome and long-term safety of these drugs have mainly been demonstrated in sponsored randomized controlled trials (RCTs) (4). However, many questions related to the prognosis and the optimal management of newly diagnosed CML patients remain unanswered, and it is widely accepted that these questions could be addressed by evaluating large prospective cohorts in real world clinical practice. Prognostic evaluation of baseline features has been reported in small single country series (5, 6) or in large datasets including 20 countries in Europe, such as the EUTOS registry (7). Until 2016, three different clinical prognostic scores were in use in clinical practice (Sokal, Euro, Eutos scores) (8), before the EUTOS Long Term survival (ELTS) score (9) was introduced, that stratifies patients in three different risk groups, with significantly different probabilities of dying of CML. The EUTOS score ability to discriminate CML patients in terms of long-term overall survival has been validated several times, but predominantly in patients treated with front-line imatinib (10–12). The score was recently suggested by the European Leukemia Net (ELN) panel in the updated recommendations (13) as a helpful tool to predict the rate of deaths related to CML in TKIs-treated patients.

The Italian CML GIMEMA network promoted an observational cohort study in January 2013, to collect a large series of consecutive newly diagnosed patients and evaluate the management and the long-term outcomes in a real-world perspective.

The aim of this article is to analyze the impact of the baseline patients’ characteristics and their front-line treatments on long term overall survival and CML-related deaths.

## Patients and Methods

The CML Italian GIMEMA network (including 68 Hematology Centers from 19 Italian regions) prospectively recorded, in a dedicated web-based database (https://www.epiclin.it/lmc), the clinical and biological features of all newly diagnosed adult (>18 years) Italian Ph+ CML patients in each phase of disease, diagnosed from January 2013 onwards. All consecutive patients were included, without any exclusion criteria and regardless of their participation in any other clinical trial, to limit the eligibility criteria selection bias typical of experimental studies. All centers followed the ELN guidelines currently available and their updates (13, 14) for the management of patients, without any other predefined recommendations, including the selection of first line TKI treatment and the subsequent monitoring every 3 months.

The study was approved by the local ethics committees and other competent authorities; all patients were registered after obtaining prior informed consent.

Standardized information on all newly diagnosed patients was collected and entered in the database by local staff and centrally checked for completeness and coherence. Baseline information included sociodemographic, clinical, and standard laboratory data. Comorbidities were evaluated by medical staff before the start of treatment according to the Charlson Comorbidity index (CCI) (15). CML was classified as chronic phase (CP), accelerated phase (AP) and blast phase (BP) according to the ELN criteria (13). Cytogenetic analysis was performed according to banding analysis at baseline, as well as qualitative molecular analysis to define the type of BCR-ABL transcript. Sokal (16) and ELTS (9) scores were calculated as previously reported. Any front-line treatment was recorded. Overall survival (OS) and cause-specific survival (CML-related deaths, other causes of death) were calculated from the date of diagnosis. Cause of death was clinically defined as leukemia-related when it occurred after progression to accelerated phase (AP) or blast phase (BP). All other deaths were classified as leukemia-unrelated, and the specific causes of death were recorded.

In this article we present the baseline characteristics (age, sex, comorbidities, prognostic scores), the type of TKI initially prescribed (Imatinib - IMA or 2^nd^ generation TKIs -2GTKI) and their impact on survival, overall and by cause of death. For OS analyses, Kaplan–Meier curves were calculated. To quantify differences in survival probabilities between groups, the log-rank test was applied. Cox proportional hazard regression models were applied to analyze the influence of all the variables considered on OS. Harrell’s C statistic was calculated to assess the discrimination ability of the two prognostic scores.

To analyze the association of prognostic factors and of first-line treatments on CML mortality, deaths due to other causes were considered competitive events. Separate cumulative incidence curves for CML and other causes mortality were estimated with the Gooley method. Adjusted sub–Hazard Ratios (sHR) for prognostic factors and treatment were estimated separately for CML and other causes mortality with the Fine and Gray method.

## Results

### Baseline Characteristics of the Enrolled Population

A cohort of 1206 patients was prospectively analyzed, 608 (50.4%) of whom received front-line IMA and 598 (49.6%) a 2GTKI (**Table 1**).

**Table 1:** Baseline CML patient characteristics by front-line TKI treatment.

Patient characteristics	First-line treatment				Total (N=1206)	
	Imatinib (N = 608)		2GTKI (N = 598)			
	N	%	N	%	N	%
Age:						
median (iqr)	69	(58-77)	52	(41-63)	60	(48-71)
mean (sd)	65.7	(14.7)	51.6	(14.0)	58.6	(16.0)
18-29	16	2.6	34	5.7	50	4.1
30-39	23	3.8	94	15.7	117	9.7
40-49	51	8.4	133	22.2	184	15.3
50-59	81	13.3	148	24.7	229	19.0
60-69	139	22.9	129	21.6	268	22.2
70-79	192	31.6	52	8.7	244	20.2
+80	106	17.4	8	1.3	114	9.5
Sex:						
Male	383	63.0	344	57.5	727	60.3
Female	225	37.0	254	42.5	479	39.7
Period of diagnosis:						
2013-2015	113	18.6	172	28.8	285	23.6
2016-2017	257	42.3	261	43.6	518	43.0
2018-2020	238	39.1	165	27.6	403	33.4
Comorbidity:						
Cardiovascular disease	238	39.1	90	15.1	328	27.2
Pulmonary disease	110	18.1	33	5.5	143	11.9
Metabolic disease	75	12.3	29	4.8	104	8.6
Other neoplasm	61	10.0	31	5.2	92	7.6
Charlson index score:						
2	359	59.0	489	81.8	848	70.3
3	104	17.1	48	8.0	152	12.6
4	80	13.2	26	4.3	106	8.8
5	46	7.6	19	3.2	65	5.4
not available	19	3.1	16	2.7	35	2.9
CML Phase:						
chronic	590	97.0	574	96.0	1164	96.5
blastic	3	0.5	2	0.3	5	0.4
accelerated	7	1.2	12	2.0	19	1.6
not available	8	1.3	10	1.7	18	1.5
ELTS score:						
Low	305	51.3	390	66.9	695	59.0
Intermediate	211	35.5	128	22.0	339	28.8
High	79	13.3	65	11.1	144	12.2
not available	13	2.1	15	2.5	28	2.3
Sokal score:						
Low	164	27.7	261	44.8	425	36.1
Intermediate	340	57.3	201	34.5	541	46.0
High	89	15.0	121	20.8	210	17.9
not available	15	2.5	15	2.5	30	2.5

The age distribution shows a clear imbalance between treatments. There was no difference of observation time between age groups. Median age in the IMA cohort was 69 years (range 58-77) versus 52 years in the 2GTKI cohort (range 41-63). In the IMA group 28% were under 60 and 49% over 70, while in the 2GTKI the corresponding figures were 68% and 10%. The male/female ratio was 1.70 in the IMA group and 1.35 in the 2GTKI cohort. Regarding the year of diagnosis, 2GTKI were prescribed more frequently in the first period (2013-2015) and less in more recent years (2018-2020). Overall, 98% of patients were in CP, versus 0.4% in blast phase and 1.6% in accelerated phase at baseline.

Results of molecular analysis of the BCR-ABL transcript at baseline showed: b2a2 in 33.1% of patients and b3a2 in 59.9%, while an atypical transcript was found in 7%. No other meaningful differences were observed according to treatments. Cytogenetic analysis at baseline showed additional cytogenetic aberrations (ACA) in 7.3% of patients (5.7% classified as major and 1.6% as minor) in the whole population. According to treatment, there were 5.3% of ACA (32/608) in the IMA cohort and 9.4% (56/598) in the 2GTKIs group respectively. According to the different type of ACA (Major and Minor route), high risk minor route ACA were detected only in 1/10 2GTKI treated patients (del7q) and in none of the IMA cohort.

In the IMA cohort, 27.7%, 57.3% and 15% of patients were stratified as low, intermediate and high risk, according to the Sokal score, whereas according to the ELTS score 51.3%, 35.5% and 13.3% of patients were classified as low, intermediate and high risk, respectively. In the 2GTKI cohort, 44.8%, 34.5% and 20.8%, were low, intermediate and high risk, according to the Sokal score, whereas according to the ELTS score, 66.9%, 22% and 11% were assigned to the respective risk groups.

The prevalence of comorbidity was at least double in the IMA group: at baseline 39% of patients presented cardiovascular comorbidities, 18% had previous pulmonary diseases and 12% a metabolic disorder. In the 2GTKI cohort, baseline cardiovascular comorbidities, pulmonary diseases and metabolic disorders were recorded in 15%, 5.5% and 4.8%, respectively. The CCI was evaluated in 82% of patients treated with IMA, and the resulting scores were 2-3 and 4-5 in 74% and 26% of patients, respectively. In patients treated with 2GTKI the CCI was available in 76% of patients and resulted 2-3 in 89.8%, and 4-5 only in 10.2%.

### Overall Survival and Cause-Specific Mortality

Overall, median follow-up of the whole population was 24.7 months (IQR: 13.3-39.3): 23.0 (10.3-37.0) and 33.2 (17.2-47.5) for the IMA and 2GTKI groups, respectively.

**Figure 1:**
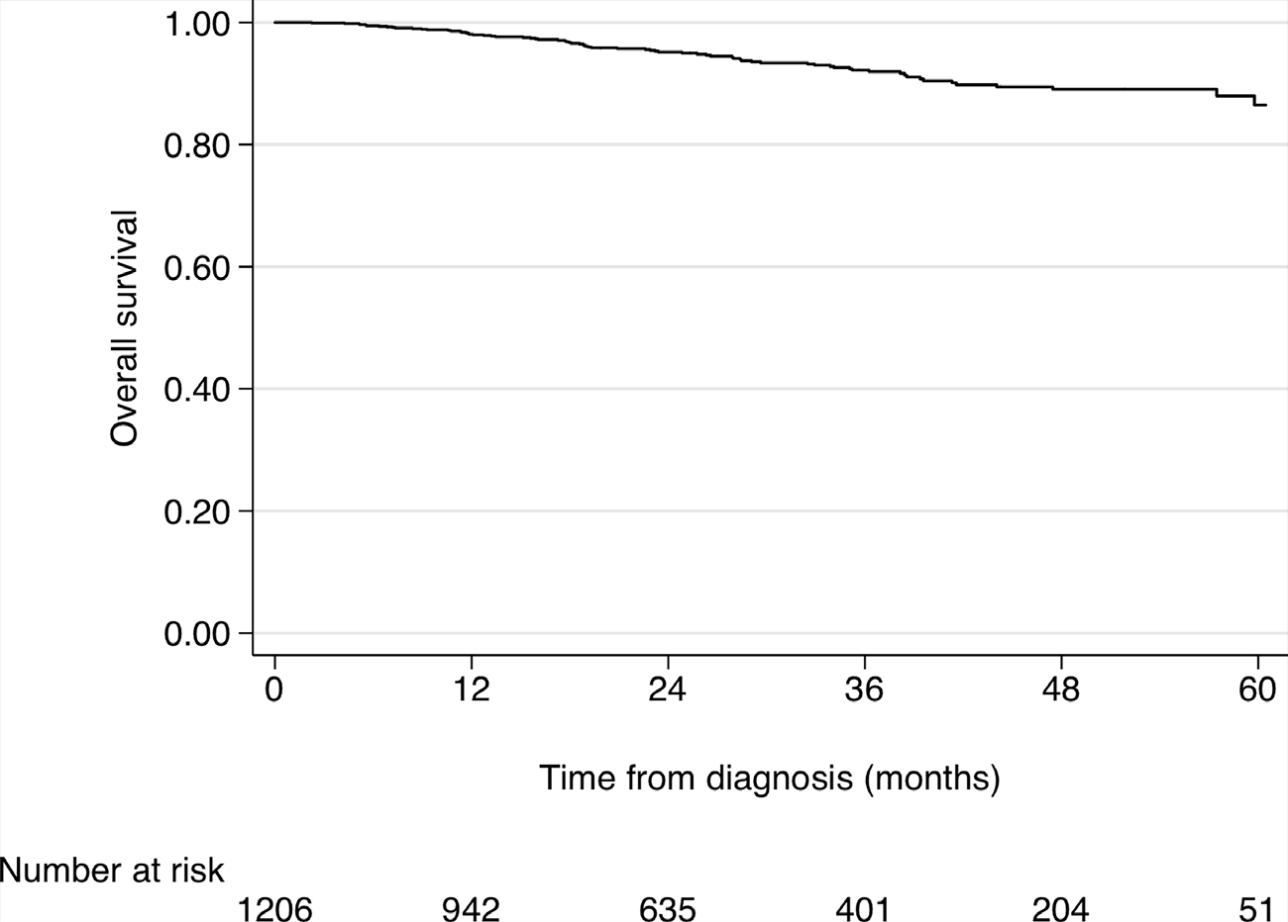
Cumulative Overall Survival of the whole CML cohort.

In the overall population, 73 patients (6.1%) died (**Table 2**). During follow-up, 56 patients (9.2% of the IMA cohort) died at a median age of 80.5 years (range 73-85), but only 11/56 (19.6%) due to CML-related causes. Indeed, 45/56 patients (80.4%) died of other causes, mostly cardiovascular diseases (19.6%) and a second neoplasia (19.6%). Conversely, in the 2GTKI cohort, only 17 patients (2.8%) died, at a median age of 62 years (range 53-72), 10/17 (58.8%) due to CML-related causes. The principal causes of death in the 2GTKI cohort were a second neoplasia (N=3) and gastro-intestinal disorders (N=2). Estimated 2- and 4-years OS of the entire cohort were 95.2% (95%CI 93.5-96.4) and 89.0% (95%CI 85.9-91.4), respectively (**Figure 1**).

**Table 2:** Causes of death of CML patients by front-line TKI treatment.

Causes of death	First-line treatment				Total (N = 73)	
	Imatinib (N = 56)		2GTKI (N = 17)			
	N	%	N	%	N	%
CML-related	11	19.6	10	58.8	21	28.8
Other causes:	45	80.4	7	41.2	52	71.2
cardiac	11	19.6	1	5.9	12	16.4
neoplasia	11	19.6	3	17.6	14	19.2
lung	3	5.4	0	0.0	3	4.1
infections	1	1.8	0	0.0	1	1.4
neurologic	5	8.9	0	0.0	5	6.8
gastroenteric	3	5.4	2	11.8	5	6.8
other/unknown	10	17.9	1	5.9	11	15.1

The crude and adjusted effects of prognostic variables and of front-line treatment on OS are reported in **Table 3**. All the variables considered showed strong and statistically clear crude effects on OS. Harrel’s C statistic, estimated to compare the discriminant propriety of the two CML risk scores, was 0.705 for the ELTS score and 0.640 for the Sokal score, confirming a better performance of the former. In the multivariable Cox model, the adjusted effects of increasing age, more comorbidities and a high ELTS risk remained clearly associated with poorer survival. Patients treated with 2GTKI showed a substantial reduction of the risk of death for any cause (HR=0.50, 95%CI 0.26-0.94) even after adjustment. When the Sokal score was analyzed in the multivariable Cox model, instead of the ELTS, its impact on OS was weaker and less precise.

**Table 3:** Role of prognostic variables and front-line treatments on overall survival of CML patients.

	Crude effects			Adjusted effects		
	HR	95% CI	p	HR	95% CI	p
Age (per year)	1.07	1.05-1.10	<0.001	1.03	1.00-1.05	0.019
Sex (F vs M)	0.59	0.35-0.97	0.040	0.71	0.42-1.21	0.211
Charlson C.I. (ref=2)						
3	2.87	1.50-5.48	0.001	1.73	0.88-3.38	0.111
>=4	6.66	3.98-11.13	<0.001	3.61	2.10-6.22	<0.001
2GTKI (ref=Imatinib)	0.22	0.13-0.39	<0.001	0.50	0.26-0.94	0.025
ELTS risk (ref=low)						
medium	3.45	1.91-6.25	<0.001	1.69	0.88-3.24	0.112
high	7.71	4.22-14.07	<0.001	4.80	2.48-9.30	<0.001
Sokal risk (*)						
medium	3.52	1.77-7.00	<0.001	1.10	0.49-2.46	0.815
high	4.22	1.96-9.08	<0.001	2.19	0.91-5.29	0.080

*Estimated in a model including all the covariates in the tables except the ELTS score. Hazard Ratio (HR) and 95% Confidence Intervals (CI) estimated with Cox regression models.

**Figure 2:**
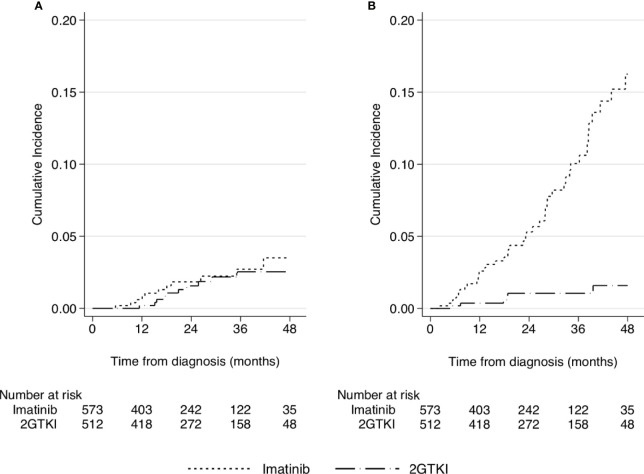
Cumulative incidence of mortality related to CML **(A)** or to other causes of deaths **(B)** treated as a competing event, according to front-line TKI treatment.

Because of the wide differences in age and distribution of comorbidities between the patients receiving the two first-line treatments, and the different causes of deaths that occurred in these groups, a comparison for CML-related deaths was performed, considering other causes of deaths as competing events. The cumulative mortality risk for CML-related causes did not show any meaningful difference between treatments (**Figure 2A**), all the difference being wholly attributable to the other causes of deaths (**Figure 2B**).

**Figure 3:**
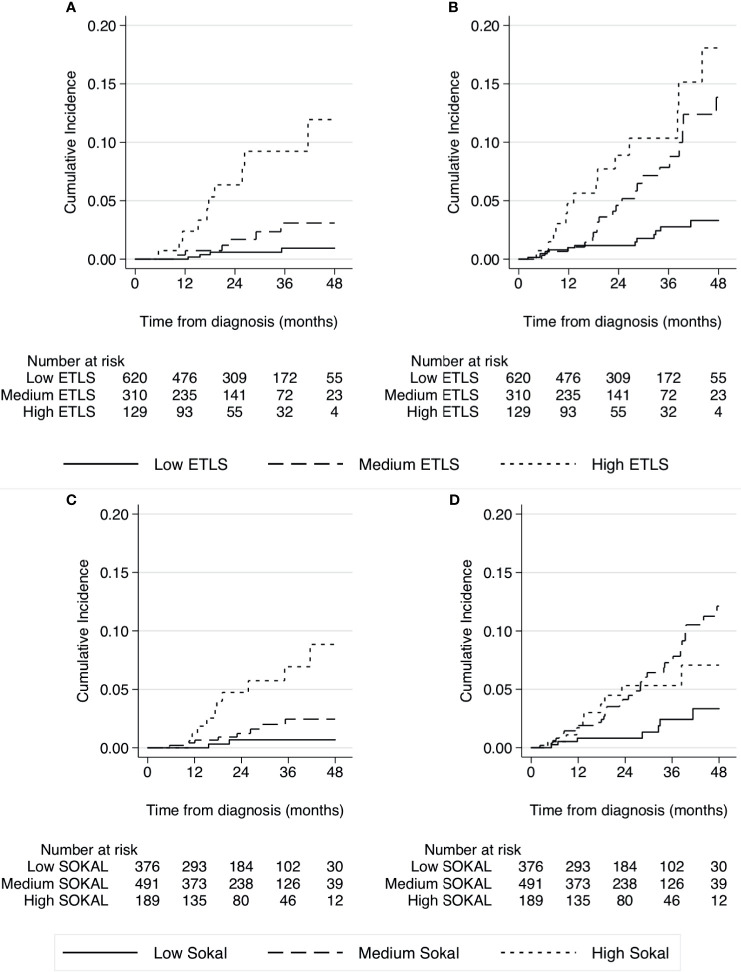
Cumulative incidence of mortality related to CML and other causes of deaths (competing event) by prognostic baseline risk, according to the ELTS **(A, B)** and Sokal score **(C, D)**.

The performance of the two CML prognostic scores is described in the graphs in **Figure 3**. The ELTS score showed a good discrimination of the three classes of risk, both for the CML-related deaths (**Figure 3A**) and for the other causes (**Figure 3B**). The discriminant ability of the Sokal score was slightly lower for CML-related causes (**Figure 3C**), but especially for the other causes of death (**Figure 3D**).

Finally, the adjusted effects of the variables of interest on the two groups for causes of deaths were estimated with a Fine and Gray model (**Table 4**). The effect of age was similar, but the CCI was a strong prognostic factor for other causes, but not for CML-related deaths. The apparent benefit of the 2GTKI on OS disappeared completely when the CML-related deaths were considered, their effect being completely attributable to a reduced risk of the other causes of deaths, and in particular of age.

**Table 4:** Role of prognostic variables and front-line treatments on survival of CML patients by cause of death (CML related or others).

	CML deaths			Other causes		
	sHR	95% CI	p	sHR	95% CI	p
Age (per year)	1.03	0.99-1.07	0.176	1.03	0.99-1.06	0.064
Sex (F vs M)	0.88	0.32-2.43	0.805	0.65	0.34-1.27	0.207
Charlson C.I. (ref=2)						
3	1.49	0.47-4.71	0.497	1.75	0.73-4.20	0.210
>=4	1.18	0.34-4.07	0.797	5.22	2.56-10.65	<0.001
2GTKI (ref=Imatinib)	1.61	0.52-4.96	0.406	0.26	0.10-0.65	0.004
ELTS risk (ref=low)						
medium	2.36	0.55-10.05	0.245	1.50	0.71-3.15	0.285
high	9.67	2.94-31.74	<0.001	3.11	1.52-6.35	0.002
Sokal risk (*)						
medium	1.76	0.34-9.11	0.498	0.83	0.33-2.08	0.688
high	6.94	1.39-34.54	0.018	1.07	0.35-3.24	0.909

*Estimated in a model including all the covariates in the tables except the ELTS score. Sub Hazard Ratio (sHR) and 95% Confidence Intervals (CI) estimated with a multivariable Fine and Gray regression model.

The performance of the two CML prognostic scores was much clearer in predicting the risk for CML-related deaths than for the other causes of deaths. For both outcomes, the ELTS showed a better discriminant ability than the Sokal score.

## Discussion

In the last decade, the availability of different first line TKIs has changed the clinical management of CML patients, allowing a better diagnostic work-up and careful evaluation of baseline comorbidities to select the best therapeutic option. In fact, the consequence of TKI-related improved survival is an increased probability of dying of other, unrelated causes: for this reason, it is of paramount importance to analyze specific causes of deaths separately, and avoid specific drug-related off-target effects.

In this study we report the overall survival and CML-related death probability of a large CML population, prospectively enrolled in a multicentric Italian observational study, together with estimates of the role of major prognostic factors and of first line treatments. Prescription preferences by Italian centers clearly showed that imatinib was prevalently chosen for older patients, whose median age was 69 years older, and who had an increased burden of comorbidities, whereas a 2GTKI was reserved to the younger and healthier population. The 4 years OS for the complete cohort was 89%, a result not so different from those reported in randomized controlled trials (RCT) of younger, more selected patients.

Previous reports analyzed large CML cohorts outside clinical trials but prevalently treated with imatinib: the EUTOS group reported a CML population collected in 20 predefined countries and regions in Europe between January 2008 and December 2013, showing an OS probability at 30 months of 92%, with a risk of dying in remission of 1% after 24 months (17). In this population, the ELTS score was tested and showed a significant difference in OS, namely 96%, 89% and 84% in the low, intermediate, and high-risk groups, respectively (8). The EUTOS group reported a subsequent validation of the ELTS score in 2949 CML patients, of whom 236 died, 89 of CML-related causes (12). The overall probability of dying of CML was 5%: applying the ELTS score both the intermediate and high-risk groups had significantly higher probabilities of dying of the disease as compared to the corresponding risk groups defined by the Sokal score. The results of our study are in line with the report by the EUTOS group but are based on a population in which about 50% of patients were treated with a 2GTKI rather than mainly with imatinib.

Since the introduction of imatinib as front-line treatment, older age appears to have lost much of its prognostic relevance. Several experiences have been reported based on age stratification: among them, the MDACC experience showed that older patients had similar cytogenetic response rates and survival compared to younger patients in chronic phase, whereas a worse survival was reported for patients in an advanced phase of the disease (18). Characteristics of CML and rates of responses vary according to age, as demonstrated in a large analysis including 2784 adult patients: the frequency of splenomegaly was more evident in younger patients, as also a high-risk stratification according to prognostic scores, and lower rates of cytogenetic responses with a higher risk of progression (19). In contrast, the German group reported that younger patients do well with imatinib despite baseline prognostic indicators (20). Age may have an influence on the initial dose and compliance to imatinib but the real impact on overall survival was related to comorbidities and to a higher risk of death from other causes, unrelated to CML, confirming the findings of a study on 181 patients aged over 75 years observed in real life practice (21). However, in the EUTOS registry, older age, more peripheral blasts, an enlarged spleen, and low platelet counts were significantly associated with an increased probability of dying of CML (8).

Comorbidities may affect survival and the choice of treatment in CML. A first observation analyzed the Charlson comorbidity index in 125 older CML patients treated with dasatinib in relation to compliance and the onset of pleural effusions, showing a direct association between a higher comorbidity score and drug-related side effects (22). The role of comorbidities and the prognostic role of Charlson index stratification on CML outcomes was further assessed by the German group: 1519 patients entered this analysis, and no differences were detected in terms of cumulative incidences of accelerated and blast phase or remission rates in the different groups. Indeed, higher scores according to the Charlson index were significantly associated with lower overall survival probabilities, with an 8-year survival of only 46.4% in patients with score >7 versus 93.6% in patients with score 2 (23). The presence of comorbidities may also be correlated with the risk of developing adverse events with TKIs. In particular, this association is highlighted by the increasing risk of arterio-occlusive events in patients with other pre-existing cardiovascular risk factors (18). As shown also in our study, in CML patients the assessment of comorbidities and the baseline age may affect TKI selection and dictates close follow-up to optimize the TKI dose.

The estimated crude effects of the variables analyzed were all strongly associated to OS, with a HR of 0.22 in favor of the 2GTKI. Even adjusting the comparison between these drugs by applying a multivariable Cox model, the protective effect of a 2GTKI on overall survival remained remarkable, with an HR of 0.50. However, the proportion of patients who died of CML-related causes was similar in the two cohorts: 1.8% in the imatinib cohort and 1.7% in the 2GTKI cohort. Furthermore, considering the CML-related deaths separately from those due to other causes, treated as competing events, no difference could be detected between treatments in terms of the cumulative risk of CML mortality: all the difference observed for OS was attributable to an increased mortality due to other causes. The analyses performed with the Fine and Gray model, that can account for competing events, clarified the role of the study variables on CML-related death or other causes death. These results did not confirm any advantage of 2GTKIs on CML mortality, in line with the results of the meta-analyses of randomized trials (4). However, these agents induced faster and deeper molecular responses but without differences in overall survival as compared to imatinib (24–26). The rationale to start a 2GTKI was supported by a subanalysis of the ENESTnd, that reported an increased rate of sustained deep molecular response with the 2GTKI when started as front-line treatment as compared to imatinib, allowing an increased proportion of patients to become candidates for a possible discontinuation over time (24). However, the optimization of the dose to reduce the possible long-term off-target events, in particular the cardiovascular side effects associated to these drugs, and the effect of treatment discontinuation, are still a matter of debate.

The principal strength of this large prospective and representative national cohort is that the TKI choice was performed according to a “patient-centered approach”, considering at baseline the prognostic role of age, concomitant comorbidities, prognostic score stratification and all the possible concomitant factors that could have influenced the adherence in the long-term (27, 28). Although the comparison between treatments has been adjusted for a set of pre-defined important prognostic factors, and the causes of death analyzed separately, the observational study design does not allow the role of uncontrolled or residual confounding to be excluded.

In conclusion, the analysis conducted showed that some specific clinical factors could be predictive of long-term overall survival in CML patients treated with TKIs. In particular, the comorbidity profile and the stratification by the ELTS score have to be considered at baseline as the mainstay on which to base decisions about the therapeutic strategy best suited to each patient. No difference between IMA and 2GTKI was observed for CML-related mortality.

## Data Availability Statement

The data analyzed in this study is subject to the following licenses/restrictions: Observational study. Requests to access these datasets should be directed to gianni.ciccone@cpo.it.

## Ethics Statement

The studies involving human participants were reviewed and approved by University of Bari. The patients/participants provided their written informed consent to participate in this study.

## Author Contributions

GSp, PP, MBr, GSag, and GCi collected, analyzed and wrote the manuscript. GCa and CM made the statistical analysis. All authors contributed to the article and approved the submitted version.

## Conflict of Interest

The authors declare that the research was conducted in the absence of any commercial or financial relationships that could be construed as a potential conflict of interest.

## Publisher’s Note

All claims expressed in this article are solely those of the authors and do not necessarily represent those of their affiliated organizations, or those of the publisher, the editors and the reviewers. Any product that may be evaluated in this article, or claim that may be made by its manufacturer, is not guaranteed or endorsed by the publisher.
